# Semi-annual Meeting of the United States Veterinary Association

**Published:** 1884-04

**Authors:** 


### SEMI-ANNUAL MEETING OF THE UNITED STATES VETERINARY ASSOCIATION.

WHOLLY informal and delightfully social was the dinner of the United States Veterinary Medical Association in one of the parlors of Young's Hotel yesterday afternoon. Forty sat at the board, nearly all members of the Association, with Dr. Fisher of the Rhode Island State Board of Health and one or two others as invited guests. The dinner was not hurried through to accommodate orators, but was eaten as befits a good dinner, at ample leisure. The Association had all day for speeches, and made them in abundance at the morning and evening business sessions of their twenty-first semi-annual meeting. The delegates represented many States, and included such well-known veterinary surgeons as Professors Liautard and Robinson of New York, Huidekofer of Philadelphia, Lyman and Harrison of Harvard, Drs. Laidlaw of Albany, Corlies of *Turf, Field and Farm*, and Bryden, Winchester, Saunders, Very, Billings and Wood of this State. Dr. Thayer of West Newton was the Nestor of the party. At the business session Dr. W. B. E. Miller of New York presided, and Professor C. B. Meechner of the same State was secretary. In addition to Professor Huidekofer, Drs. E. Burket and B. L. James were elected new members and nineteen propositions for membership were received. Ten of these were from the Western States.

Dr. Liautard reported upon the congress at Brussels in September, to which he was a delegate, and papers were read on the progress of veterinary associations and on important kinds of veterinary surgery.

After dinner Dr. McLean of Brooklyn had a brief discussion on the extent to which certain zymotic diseases in cattle rendered the meat unfit for use as food, and whether the test of the glands of the groin and axilla being affected was a sufficient one. Dr. Bailey of Portland, Me., then brought up the subject of eczema contagiosa, or foot and mouth disease. There are, he said, five quarantine stations on farms outside of Portland where infected animals have been, and these farms contain about 200 tons of hay and a quantity of manure. What is to be done with that hay? Can it be disinfected so as to be fit for animals to eat, or should it be burned? Can the manure be used with safety? Dr. Corlies thought the manure might be ploughed in and the hay used for cattle in quarantine, but not if they were to mix with healthy cattle. He thought the best plan would be to burn it. This opinion was endorsed by others, who held further that the manure should also be destroyed, and any loss sustained should be paid by the United States Government.

Professor Huidekofer took the ground that the Government was directly responsible. They imported cattle which they professed had been inspected, and these were driven over public roads, and left the disease there, and infected the hay on the farms where they were kept. Dr. Bailey said that on February 2, twenty-eight cattle were landed from the steamer Ontario, supposed to be free from disease. They were driven three miles over a public road to the quarantine farms. A man with some oxen belonging near by followed them, and a few days later the imported cattle were found to have the foot and mouth disease, and soon after the oxen were also seized with it, thus showing the source of infection. From those oxen every case of the disease in that part of the country can be traced. He censured the system of inspection.

Drs. Corlies, Harrison and Lyman also took part in the discussion. The latter thought the association should give an expression of opinion on the very careless system of inspection.

After further discussion, Prof. Huidekofer moved a resolution, which was adopted, that, in the opinion of the association, the manure and hay on the infected premises at Portland should be destroyed; that the United States quarantine authorities, through carelessness and incompetency, are responsible for the spread of the infection, and responsible in a monetary sense for the loss to the citizens; also that the roads traversed by the animals with the disease should be disinfected. Dr. Billings supported this vigorously, urging the importance of compelling the government to recognize the veterinary profession.

Dr. Bryden spoke of the inefficiency of the inspection at Boston, and considered the commissioners grossly negligent. He complained that the whole sanitary system was at fault and required revision, but that political influence prevented. He thought it time that the commissioners should admit their failure and resign. Dr. McLean moved in addition that the veterinary profession was not properly represented in the quarantine system, as there are inspectors of ports who are not veterinary surgeons. This was adopted.

Dr. Winchester of Lawrence related his experience with three cases of actinomycosis, or big head, in the past year, and a discussion followed on the increase of this formerly rare disease, and as to its being confounded with tuberculosis. Dr. Billings considered the disease invasive rather than infectious. Dr. Bunker made inquiries as to the experience of the Association in cases of horses affected by the use of ensilage or the proximity of silos to their stalls.

Dr. Billings spoke of several fatal cases of paralysis of the throat, which he thought due to this cause, but the president secretary and others thought the cases must be a form of cerebro-spinal meningitis, not due to ensilage, the use or proximity of which they considered harmless to horses. Dr. Billings did not agree with them.

Notice of a motion to increase the number of censors from five to seven was given for the next session.

The question of the place at which the next meeting will be held caused a long debate on the respective merits of Cincinnati and Philadelphia. After many motions and amendments the matter was left with the commissa minora, with power to decide.

During the evening fifteen Massachusetts graduates met to form a State veterinary society. Dr. F. Osgood of Springfield presided, and Dr. Bunker of Newton acted as secretary. Drs. Bryden, Saunders, Lyman, Billings and Howard were appointed a committee to examine credentials and meet on a future evening.—*Boston Globe.*

